# Molecular Characterization of Carbapenem-Resistant *Enterobacter cloacae* in 11 Chinese Cities

**DOI:** 10.3389/fmicb.2018.01597

**Published:** 2018-07-17

**Authors:** Chunmei Jin, Jiangang Zhang, Qi Wang, Hongbin Chen, Xiaojuan Wang, Yawei Zhang, Hui Wang

**Affiliations:** ^1^Department of Clinical Laboratory, Yanbian University Hospital, Yanji, China; ^2^Department of Clinical Laboratory, Peking University People's Hospital, Beijing, China

**Keywords:** carbapenem-resistance, *Enterobacter cloacae*, carbapenemase, NDM-1, ST418

## Abstract

Carbapenem-resistant *Enterobacteriaceae* (CRE) are usually resistant to most of antibiotics. Infections caused by such bacteria have a high mortality and pose a serious threat to clinical management and public health. *Enterobacter cloacae* ranks third among *Enterobacteriaceae* that cause nosocomial infections. In this study, the molecular characteristics of carbapenem-resistant *E. cloacae* in China were investigated. From November 2012 to August 2016, 55 non-repetitive strains of carbapenem-resistant *E. cloacae* were collected from 12 hospitals in 11 Chinese cities. The bacteria were identified with matrix-assisted laser desorption/ionization time of flight mass spectrometry. Antimicrobial susceptibility tests were determined by agar dilution method. Carbapenemase and other β-lactamase genes were detected with PCR and sequencing. Multilocus sequence typing and plasmid conjugation tests were performed. Among the 55 *E. cloacae* strains, 50 strains were detected to produce 8 types of carbapenemase including NDM-1, NDM-5, IMP-4, IMP-26, IMP-1, KPC-2, and VIM-1. NDM-1 accounted for 68.0% (34/50) among the carbapenemase-producing *E. cloacae*. A total of 24 sequence types were identified and ST418 was the most common, accounting for 20% (11/55). For further investigation, a pulsed-field gel electrophoresis (PFGE) assay was conducted to identify the PFGE patterns of the strains. These 23 isolates yielded 13 PFGE patterns, which were designated as type A–M. Eight isolates obtained from Shenzhen had the same PFGE pattern (type A) and the remaining 15 isolates belonged to the other 12 PFGE patterns (type B–M). The observation that 8 of the 15 *bla*_NDM−1_-positive *E. cloacae* isolates obtained from Shenzhen with the same PFGE pattern (type A) suggested a transmission outbreak of a common strain. S1-nuclease PFGE and Southern blotting were also conducted to estimate the size of plasmids harbored by *bla*_NDM−1_-positive strains. The results showed that the plasmids harboring the *bla*_NDM−1_ gene ranged in size from approximately 52–58 kilobases. Our study indicates that carbapenem-resistant *E. cloacae* strains that produce NDM carbapenemase have strong resistance. Early detection and monitoring of the prevalence of these strains are urgent.

## Introduction

In recent years, the emergence of carbapenem-resistant *Enterobacteriaceae* (CRE) has become a serious issue both on community-acquired infections and healthcare-associated infections (van Duin and Doi, 2017). As well as other *Enterobacteriaceae, Enterobacter cloacae* (*E. cloacae*) is a conditional pathogen found in the intestine. Healthcare-associated infections caused by *E. cloacae* ranked third among all the *Enterobacteriaceae* (Dai et al., 2013). *Enterobacter cloacae* can produce chromosome mediated AmpC β-lactamase and has resistance to ampicillin, amoxicillin/clavulanic, cephamycin and first and second generation cephalosporin. A wide spectrum of antibacterial drugs such as carbapenems may be used in treatment more often. Thus, multidrug resistance has emerged rapidly under antibiotic selection pressure. Carbapenem-resistant *E. cloacae* infections have been reported in many countries such as Spain, Australia, the United States, India, and China (Kiedrowski et al., 2014; Fernández et al., 2015; Liu et al., 2015; Sidjabat et al., 2015). The emergence of carbapenem-resistant *E. cloacae* is an enormous challenge to clinical treatment. It is well known that the main mechanism for reduced susceptibility to carbapenems in *E*. *cloacae* is the deregulation of ACT (the natural cephalosporinase of *E*. *cloacae*), which is associated with a decrease in membrane permeability. In addition to this, producing carbapenemases is another important mechanism of *Enterobacteriaceae* in carbapenem resistance (Walsh et al., 2005; Nordmann et al., 2009; Tzouvelekis et al., 2012). Also, the mechanism of combinations of either ESBL or AmpC and mutation of porins may hold a certain proportion (Yang et al., 2010).

Up until now, there was a lack of multicenter research on carbapenem-resistant *E. cloacae* in China. So, we conducted this molecular epidemiological study on carbapenem-resistant *E. cloacae* to further understand the prevalence of the bacteria in China.

## Materials and methods

### Sample collection

**Figure 1:**
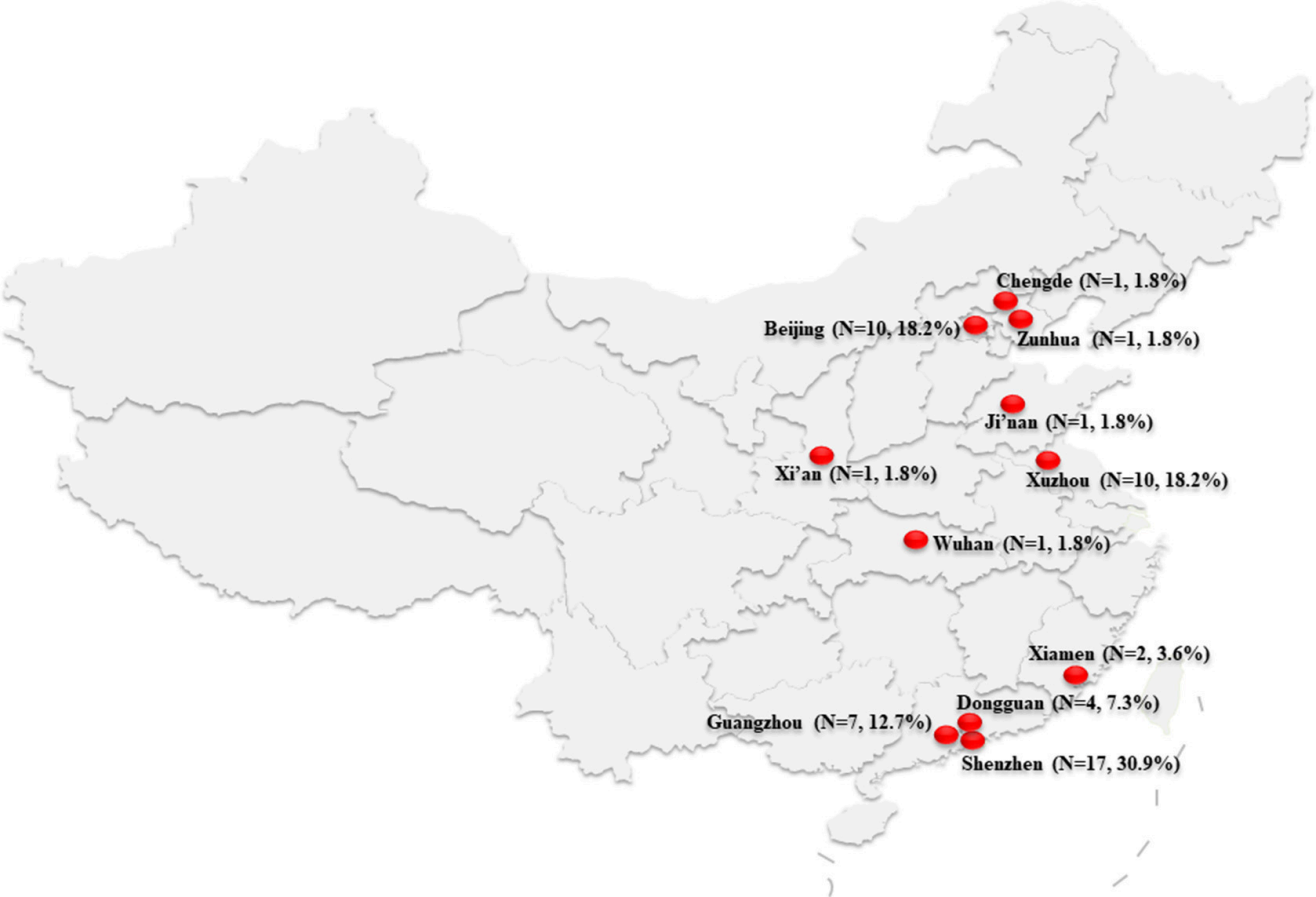
Map of China showing the location of the 11 cities where the carbapenem-resistant *Enterobacteriaceae cloacae* isolates were collected.

From November 2012 to August 2016, we collected 55 unrepeated strains of carbapenem-resistant [any carbapenem (imipenem, meropenem, or ertapenem) as determined by standard methods] *E. cloacae* from 12 hospitals in 11 Chinese cities (Beijing, Chengde, Zunhua, Ji'nan, Xuzhou, Xi'an, Wuhan, Xiamen, Guangzhou, Dongguan, and Shenzhen; Figure 1). The participating hospitals include Peking University People's Hospital, Peking Union Medical College Hospital, Affiliated Hospital of Chengde Medical University, People's Hospital of Zunhua, Qilu Hospital of Shandong University, Affiliated Hospital of Xuzhou Medical University, Xijing Hospital, Tongji Hospital, The First Affiliated Hospital of Xiamen University, The First Affiliated Hospital Sun Yat-sen University, Donghua Hospital Sun Yat-sen University, and Shenzhen Second People's Hospital.

### Identification of the bacterial strains and antimicrobial susceptibility tests

All isolates were identified with matrix-assisted laser desorption/ionization time of flight mass spectrometry (MALDI-TOF MS) (Bruker Daltonics, Bremen, Germany). Minimum inhibitory concentrations (MICs) were determined by the agar dilution method according to CLSI guidelines (M100-S27). The tested drugs included ceftriaxone (Roche China, Shanghai, China), cefotaxime, ceftazidime, cefepime, aztreonam, amikacin, levofloxacin, minocycline, fosfomycin (National Institute for Food and Drug Control of China, Beijing, China), piperacillin/tazobactam, tigecycline (Pfizer, NY, USA), imipenem (Merck Sharp & Dohme, Hangzhou, China), meropenem (Sumitomo Pharmaceuticals, Suzhou, China), ciprofloxacin (Bayer, Leverkusen, Germany), and polymyxin B (Amresco, Solon, USA). Strains used in quality control were *Escherichia coli* ATCC 25922 and *Pseudomonas aeruginosa* ATCC 27853. The results were interpreted according to 2017 CLSI standards (M100-S27). The tigecycline test was performed according to the Food and Drug Administration standards.

### Detection of antimicrobial resistance genes

Phenotypic screening for the resistance genes of carbapenem-resistant *E. cloacae* strains was based on the 2017 CLSI guidelines. Modified Hodge test (MHT), imipenem-EDTA double-disk synergy test (DDST) (Lee et al., 2001), and modified carbapenem inactivation method (mCIM) were used to test carbapenemase production. Polymerase chain reaction (PCR) was used to detect carbapenemase genes (*bla*_NDM_, *bla*_KPC_, *bla*_IMP_, *bla*_IMI_, *bla*_NMC_, *bla*_GES_, *bla*_SME_, *bla*_SIM_, *bla*_VIM_, and *bla*_OXA−48_) and other β-lactamase genes (*bla*_CTX−M_, *bla*_TEM_, *bla*_SHV_, *bla*_DHA_, and *bla*_CMY_) (Lewis et al., 2007; Yang et al., 2010). The products were submitted for sequencing.

### Multilocus sequence typing (MLST)

MLST was performed according to a previously described method (https://pubmlst.org/ecloacae/). New alleles and sequence types were submitted to the MLST website and approved. Sequence Type Analysis and Recombinational Tests 2 (START2) (http://pubmlst.org/software/analysis/start2/) software was used to generate the phylogenetic tree (Jolley et al., 2001).

### Plasmid conjugation test

The plasmid conjugation test was used to test carbapenem-resistant gene transfer. Ten strains were selected for the test. *Escherichia coli* EC600 (rifampicin resistant) was used as the recipient. Conjugants were screened using China blue lactose agar plates containing rifampicin (300 μg/ml) and imipenem (1 μg/ml). The donor and the recipient were mixed at a ratio of 1:1 for 24 h. Transconjugants were selected on China blue lactose agar plates (OXOID, Basingstoke Hampshire, UK), supplemented with rifampicin (100 μg/ml) and imipenem (1 μg/ml). PCR was used to screen for *bla*_NDM−1_, *bla*_VIM−1_, *bla*_KPC−2_, and *bla*_IMP−1_ as previously described (Wang et al., 2014).

### Pulsed-field gel electrophoresis (PFGE)

*Enterobacter cloacae* isolates were characterized by PFGE according to the previously published protocol by Ribot et al., with modifications (Ribot et al., 2002). We selected 23 *bla*_NDM−1_-positive isolates (including the 15 isolates from Shenzhen, 6 isolates involved in the conjugation experiments, and another 2 representative isolates).

Electrophoresis conditions were altered to have an initial switch time of 2.16 s and a final switch time of 54.17 s, and gels were run for 18 h. The resulting PFGE patterns were analyzed in BioNumerics software (Applied Maths, Austin, TX, USA) with dendrograms based on the Dice coefficient with a band position tolerance of 1%. Patterns with no discernible differences were considered indistinguishable and given the same PFGE pattern designation.

### S1-nuclease PFGE and southern blotting

S1-nuclease PFGE and Southern blotting were performed to estimate the size of plasmids harbored by *bla*_NDM−1_-positive strains as described previously. We selected 23 *bla*_NDM−1_-positive isolates as mentioned above.

The *bla*_NDM−1_ gene was detected by digoxigenin-labeled specific probes (DIG High Prime DNA Labeling and Detection Starter Kit II, Roche Diagnostics, Mannheim, Germany). *Salmonella enterica* H9812 was used as a size marker.

### Statistical analyses

WHONET (version 5.6) software (http://www.whonet.org/software.html) and SPSS (version 22.0) software (SPSS Inc., Chicago, IL, USA) were used for statistical analyses.

### Ethical approval

This study was approved by the research ethics board at Peking University People's Hospital. Informed consent was not needed as this study was retrospective and participants were anonymized. Medical records and patient's information were retrospectively reviewed and collected.

## Results

### Characteristics of collected samples

The most common specimens were respiratory tract (20 cases, 36.4%), followed by urine (13 cases, 23.6%), blood (11 cases, 20%), ascitic fluid (7 cases, 12.7%), bile (2 cases, 3.6%), catheter (1 case, 1.8%), and wound (1 case, 1.8%).

### Antimicrobial susceptibility tests

Of all the antimicrobials tested, the most susceptible antimicrobial was polymyxin B (100%, 55/55), followed by amikacin (89.1%, 49/55), fosfomycin (80%, 44/55), tigecycline (78.2%, 43/55), minocycline (52.7%, 29/55), levofloxacin (30.9%, 17/55), and ciprofloxacin (25.5%, 14/55). All of the 36 *bla*_NDM_-positive strains were resistant to piperacillin-tazobactam, ceftazidime, cefotaxime, ceftriaxone, cefepime, imipenem, and meropenem. It is worth noting that the most susceptible antimicrobials to *bla*_NDM_-positive strains was polymyxin B (100%, 36/36), followed by amikacin (86.1%, 31/36), and fosfomycin (86.1%, 31/36). There was some differences between *bla*_NDM_-positive strains and strains with other carbapenemases (Table 1).

**Table 1:** *In vitro* activities of antimicrobial agents against carbapenemase-producing *Enterobacteriaceae*.

**Antimicrobials**	**All isolates (*****n*** = **55)**			**Isolates with** ***bla***_**NDM**_ **(*****n*** = **36)**			**Isolates with other carbapenemase genes except** ***bla***_**NDM**_ **(*****n*** = **14)**			**Comparison between the two groups**	
	**%S**	**MIC_50_**	**MIC_90_**	**%S**	**MIC_50_**	**MIC_90_**	**%S**	**MIC_50_**	**MIC_90_**	**χ^2^**	***P-*value**
		**(μg/ml)**	**(μg/ml)**		**(μg/ml)**	**(μg/ml)**		**(μg/ml)**	**(μg/ml)**		
Piperacillin/tazobactam	20	256	>256	0	>256	>256	64.3	8	128	25.451	< 0.001
Ceftazidime	1.8	>256	>256	0	>256	>256	7.1	256	>256	–	0.265
Cefotaxime	1.9	>256	>256	0	>256	>256	7.1	64	>256	–	0.286
Ceftriaxone	1.9	>256	>256	0	>256	>256	7.1	32	>256	–	–
Cefepime	1.8	64	128	0	128	128	7.1	8	64	–	–
Aztreonam	17.4	256	>256	6.7	256	>256	50	4	256	6.003	0.014
Imipenem	12.7	8	32	0	8	32	42.9	2	4	–	0.001
Meropenem	16.4	8	32	0	8	64	57.1	1	4	–	0.019
Amikacin	89.1	4	>256	86.1	4	>256	100	2	8	0.781	0.377
Ciprofloxacin	25.5	32	128	13.9	32	128	50	1	128	–	0.011
Levofloxacin	30.9	16	128	22.2	32	128	50	2	32	1.704	0.192
Fosfomycin	80	16	128	86.1	8	128	75	16	256	–	0.19
Minocycline	52.7	16	128	30.6	32	128	57.1	4	32	4.276	0.039
Polymyxin B	100	0.125	0.25	100	0.125	0.25	100	0.125	0.25	–	–
Tigecycline	78.2	1	8	72.2	1	8	91.7	1	1	0.838	0.36

### Genotype analysis

Among the 55 strains, 50 were confirmed to produce 8 types of carbapenemases including NDM-1, NDM-5, IMP-4, IMP-26, IMP-1, KPC-2, and VIM-1. The corresponding numbers of the strains that produced the foregoing types of carbapenemases were 34, 2, 6, 3, 2, 2, and 1. Other carbapenemase genes were not detected. No strains contained two or more carbapenemase genes. NDM-1-producing carbapenem-resistant *E. cloacae* was primarily distributed in Shenzhen (Table 2). Carbapenemase genes were not detected in the other 5 strains.

**Table 2:** Microbiological and molecular characteristics of 34 *bla*_NDM−1_-positive *Enterobacter cloacae* strains.

**Isolate**	**Date of isolation**	**City**	**Gender/Age (Year)**	**Ward**	**Specimen**	**mCIM (mm)**	**MHT**	**EDTA-DDST**	**DHA**	**CTX-M**	**ST**	**PFGE pattern**	**Plasmid size, harboring *bla*_NDM_ (kb)**
ecl408	2015/6/8	Dongguan	M/24	ICU	ur	6	+	+	–	–	418	–	–
ecl409	2015/3/28	Dongguan	M/47	ICU	ur	6	+	+	–	–	418	–	–
ecl411	2015/6/25	Dongguan	F/49	ICU	ur	6	+	+	DHA-1	–	418	–	–
cas471	2015/12/28	Zunhua	M/36	ICU	ur	6	+	+	–	CTX-M-3	920	F	~54
ecl497	2015/6/16	Ji'nan	F/59	Outpatient	sp	6	+	+	DHA-1	CTX-M-3	51	G	~52
ecl645	2014/5/23	Guangzhou	F/66	Neurology	ur	6	+	+	–	CTX-M-3	93	–	–
ecl759	2015/1/28	Shenzhen	F/60	Hepatobiliary surgery	dr	6	±	+	DHA-1	–	88	B	~52
ecl760	2015/2/6	Shenzhen	F/60	Hepatobiliary surgery	wd	6	+	+	DHA-1	–	88	B	~52
ecl766	2015/5/22	Shenzhen	M/77	Respiratory	ca	6	+	+	DHA-1	–	93	C	~52
ecl767	2015/5/22	Shenzhen	M/77	Respiratory	bl	6	+	+	–	–	93	C	~52
ecl768	2015/6/8	Shenzhen	M/47	Neurosurgery	sp	6	+	+	–	–	418	A	~52
ecl771	2015/6/18	Shenzhen	F/45	Neurosurgery	sp	6	+	+	–	–	418	A	~52
ecl774	2015/8/28	Shenzhen	F/61	Neurosurgery	ur	6	+	+	–	–	418	A	~52
ecl776	2015/9/14	Shenzhen	M/38	Neurosurgery	ur	6	+	+	–	–	418	A	~52
ecl777	2015/10/20	Shenzhen	M/34	Neurosurgery	sp	6	+	+	–	–	93	D	~52
ecl778	2015/11/22	Shenzhen	M/83	Neurosurgery	ur	6	+	+	–	–	93	D	~52
ecl779	2015/12/8	Shenzhen	F/42	EICU	sp	6	+	+	–	–	418	A	~52
ecl780	2015/12/21	Shenzhen	M/70	Neurosurgery	sp	6	+	+	–	–	93	E	~54
ecl782	2015/12/18	Shenzhen	F/42	EICU	ur	6	+	+	–	–	418	A	~52
ecl784	2015/12/25	Shenzhen	M/84	Nephrology	bl	6	+	+	–	–	418	A	~52
ecl786	2016/1/8	Shenzhen	F/42	EICU	ba	6	+	+	–	–	418	A	~52
ecl828	2015/1/11	Xuzhou	M/55	EICU	sp	6	+	+	DHA-1		51	–	–
ecl830	2015/1/23	Xuzhou	M/40	Neurosurgery	sp	6	+	+	–	CTX-M-3	51	–	–
ecl844	2015/12/22	Xuzhou	M/77	EICU	sp	6	+	+	–	CTX-M-3	51	H	~52
ecl886	2015/5/8	Xiamen	M/85	ICU	ab	6	+	+	–	–	171	I	~52
ecl932	2016/5/27	Xiamen	M/61	Urology Surgery	ur	6	+	+	–	–	78	–	–
ecl979	2016/6/26	Wuhan	M/46	ICU	ur	6	+	+	–	–	78	J	~58
ecl982	2016/5/31	Xi'an	F/57	Hepatobiliary surgery	dr	6	+	+	–	–	78	–	–
ecl1017	2016/7/3	Beijing	F/35	Respiratory	bl	6	+	+	–	–	121	K	~56
ecl1028	2016/8/28	Beijing	F/59	Hematology	sp	6	+	+	–	–	127	–	–
ecl1045	2016/4/26	Xuzhou	M/59	Urology Surgery	bl	6	+	+	–	CTX-M-3	78	–	–
ecl1102	2016/6/22	Xuzhou	M/68	ICU	bl	6	+	+	–	–	231	–	–
ecl1115	2016/6/13	Xuzhou	M/74	ICU	sp	6	+	+	–	CTX-M-3	97	L	~52
ecl1127	2016/7/27	Xuzhou	M/50	EICU	sp	6	+	+	DHA-1	CTX-M-14	97	M	~55

*ecl, Enterobacter cloacae; cas, Enterobacter asburiae; ICU, intensive care unit; EICU, emergency intensive care unit; ba, broncho-alveolar lavage; bl, blood; ca, catheter; dr, drainage; sp, sputum; ur, urine; wd, wound; EDTA-DDST, EDTA double-disk synergy test*.

NDM-1-producing *E. cloacae* isolates were mainly collected from Shenzhen (44.1%, 15/34), followed by Xuzhou (20.6%, 7/34), Beijing, Dongguan, Guangzhou, Ji'nan, Xi'an, Xiamen, Wuhan, and Zunhua. These samples were primarily collected from the Intensive Care Unit and the Emergency Intensive Care Unit (41.2%, 14/34), followed by the department of neurosurgery (23.5%, 8/34). NDM-1-producing *E. cloacae* isolates were most commonly identified in sputum samples (35.3%, 12/34), followed by urine samples (32.4%, 11/34). All strains were positive for MHT, imipenem-EDTA-DDST, and mCIM. Results of the three tests were consistent. In addition, 6 strains also produced the AmpC enzyme DHA-1, 8 strains produced CTX-M-3, and 2 strains produced both CTX-M and DHA-1.

### MLST

**Figure 2:**
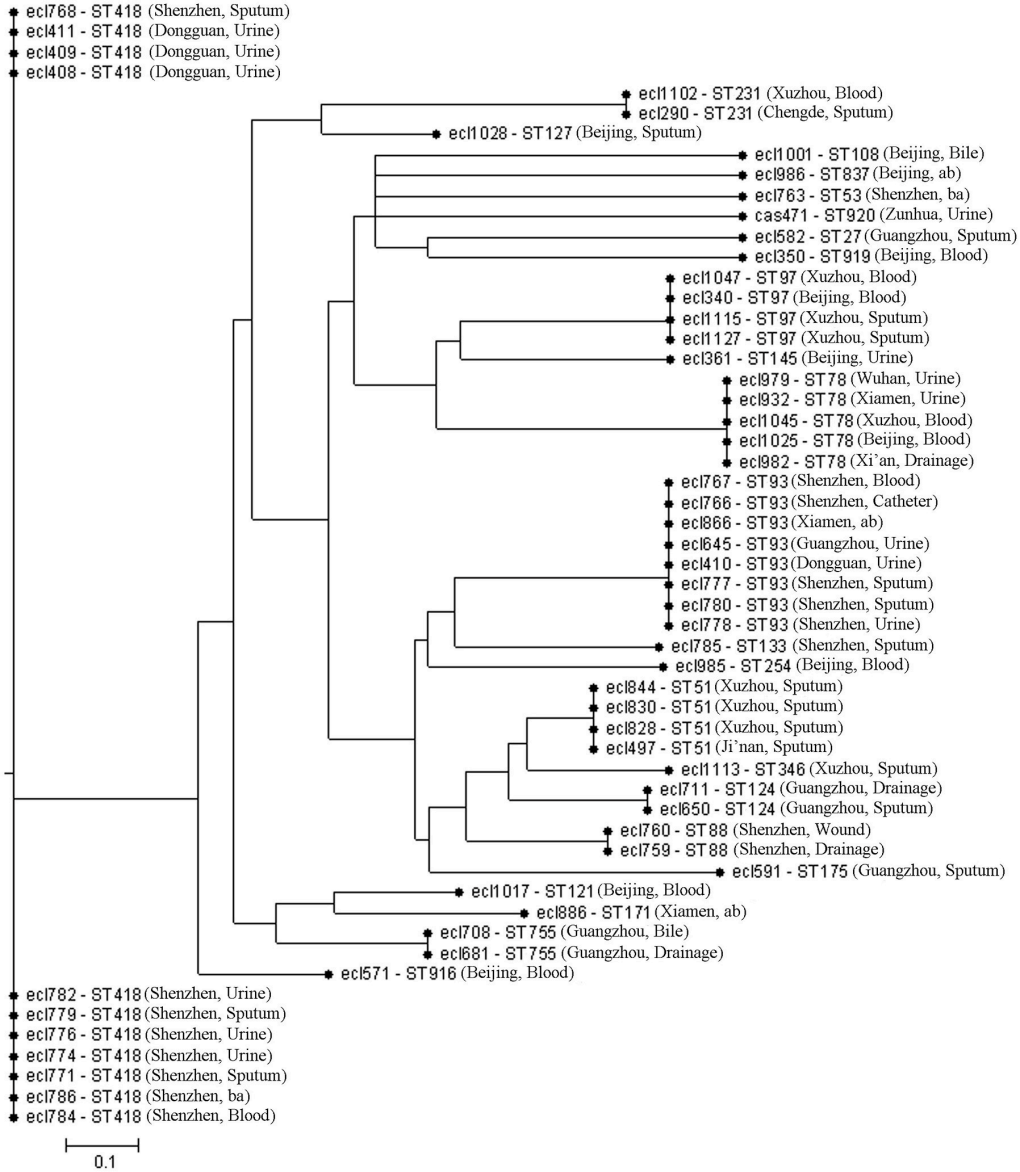
Multilocus sequence typing (MLST) phylogenetic tree of the 55 carbapenem-resistant *E. cloacae* strains. ab, abdominal fluid; ba, broncho-alveolar lavage.

The results of the MLST are shown in Figure 2. A total of 24 sequence types were detected in the 55 *E. cloacae* strains. ST418 was the most common (20%, 11/55), followed by ST93 (14.5%, 8/55).

### Plasmid conjugation test

Plasmids from 9 carbapenemase-producing *E. cloacae* strains were successfully transferred to *E. coli* EC600. Drug sensitivity tests showed the MICs of meropenem increased by 5- to 8-fold in the 9 conjugators; for imipenem, cefepime, ceftazidime, and piperacillin/tazobactam, the MICs increased by 3~6-, 7~11-, 7~10-, and 3~7-fold, respectively (Table 3).

**Table 3:** Antibiotic susceptibilities of *E. cloacae* isolates and their transconjugants (μg/ml).

**Isolate**	**City**	**Carbapenemase**	**ST**	**MEM**	**IMP**	**FEP**	**CAZ**	**TZP**	**ATM**	**AMK**	**CIP**	**LVX**	**PB**	**TGC**
***E. cloacae*** **isolates**														
cas471	Zunhua	NDM-1	920	8	16	64	>256	>256	256	8	16	16	0.25	1
ecl497	Ji'nan	NDM-1	51	8	4	32	>256	256	>256	>256	2	2	0.25	1
ecl591	Guangzhou	VIM-1	175	0.5	4	8	256	128	0.032	1	< = 0.016	< = 0.016	0.125	0.5
ecl763	Shenzhen	KPC-2	53	0.5	4	4	8	256	128	1	4	8	0.25	0.5
ecl844	Xuzhou	NDM-1	51	2	8	16	>256	128	128	4	0.25	0.5	0.125	0.5
ecl886	Xiamen	NDM-1	171	2	4	32	>256	256	128	1	2	2	0.5	1
ecl979	Wuhan	NDM-1	78	>32	>32	>256	>256	>256	–	1	>64	64	0.25	4
ecl1017	Beijing	NDM-1	121	8	8	64	>256	>256	–	2	64	16	0.125	0.5
ecl1025	Beijing	IMP-1	78	2	2	32	>256	8	–	0.5	32	32	0.125	0.25
***E. coli*** **transconjugant strains**														
471TC		NDM-1		2	8	32	>256	256	128	1	0.125	0.5	0.25	0.125
497TC		NDM-1		4	8	32	>256	128	128	1	0.125	0.25	0.125	0.125
591TC		VIM-1		2	4	128	>256	>256	0.25	1	0.125	0.25	0.125	0.125
763TC		KPC-2		4	4	8	32	>256	>256	1	2	2	0.125	0.125
844TC		NDM-1		2	8	16	>256	128	64	1	0.125	0.25	0.125	0.25
886TC		NDM-1		2	8	16	>256	128	128	1	0.125	0.25	0.125	0.25
979TC		NDM-1		4	8	16	>256	128	4	0.125	2	4	0.125	2
1017TC		NDM-1		8	16	128	>256	256	>256	2	0.125	1	0.125	0.125
1025TC		IMP-1		1	2	32	>256	16	0.125	1	0.125	0.25	0.25	0.125
EC600		–		0.032	0.25	0.064	0.25	2	0.125	1	0.125	0.25	0.25	0.25

*MEM, meropenem; IMP, imipenem; FEP, cefepime; CAZ, ceftazidime; TZP, piperacillin/tazobactam; ATM, aztreonam; AMK, amikacin; CIP, ciprofloxacin; LVX, levofloxacin; PB, polymyxin B; TGC, tigecycline; TC, transconjugant strain; EC600, recipient strain*.

### PFGE, S1-nuclease PFGE and southern blotting

When typed by PFGE to determine if they were related, the 23 isolates yielded 13 PFGE patterns, which were designated as type A–M. Eight isolates (ecl768, ecl771, ecl774, ecl776, ecl779, ecl782, ecl784, and ecl786) obtained from Shenzhen had the same PFGE pattern (type A) and the remaining 15 isolates belonged to the other 12 PFGE patterns (type B–M) (Table 2). The observation that 8 of the 15 *bla*_NDM−1_-positive *E. cloacae* isolates with the same PFGE pattern (type A) and the same sequence type (ST418) suggested a transmission outbreak of a common strain.

The results of S1-nuclease PFGE and Southern blotting showed that the plasmids harboring the *bla*_NDM−1_ gene ranged in size from approximately 52–58 kilobases, respectively (Table 2). The plasmids harboring the *bla*_NDM−1_ gene of the 8 isolates obtained from Shenzhen were the same size (approximately 52 kilobases).

## Discussion

Carbapenemase-producing *E. cloacae* has been reported in many countries, such as strains producing OXA-48 and VIM-1 have been reported in Spain. In Brazil, Australia and America, strains producing NDM-1, IMP-4, and KPC-3, respectively, have been reported (Kiedrowski et al., 2014; Rozales et al., 2014; Villa et al., 2014; Fernández et al., 2015; Sidjabat et al., 2015), while in Chongqing, Henan and Ningxia of China, strains producing NDM-1 have been identified (Dai et al., 2013; Liu et al., 2015; Shi et al., 2017). Strains that produced other carbapenemases have also been reported in the Sichuan province of China (Huang et al., 2015). In the present study, we found that the *E. cloacae* prevalent in China mainly produced NDM-1 (68.0%, 34/50) and IMP-4 (12.0%, 6/50). NDM-1 was found in the highest proportion and may represent a significant drug-resistant mechanism of carbapenem-producing *Enterobacteriaceae* in China.

The plasmid conjugation test was completed with 6 *bla*_NDM−1_-positive strains. Conjugants were all detected to have the *bla*_NDM−1_. Susceptibility results showed that compared with the receptor bacteria EC600, the conjugants have a higher MIC value on cephalosporins and carbapenems. There were no MIC promotions on polymyxin B and tigecycline between conjugants and EC600. Many studies have demonstrated that the plasmid owned *bla*_NDM_ also have other resistant genes, such as *bla*_TEM−1_, *bla*_CMY_, qnrA6, and qnrB1 for quinolone resistance, armA, rmtA, and rmtC for aminoglycoside resistance (Poirel et al., 2011a,b; Kocsis et al., 2016). But all strains in this study have no *bla*_TEM−1_ and *bla*_CMY_. Five of the conjugants have no MIC difference on quinolone with the EC600. Maybe the relative plasmid did not harbor the quinolone resistant gene.

MLST showed subtype diversity. A total of 24 sequence types were detected in 55 *E. cloacae* strains. ST418 was detected the most frequently (11/55, 20%), and the second was ST93 (14.5%, 8/55). Three new sequence types were found, namely ST916, ST919, and ST920. Our study reveals the diversity of carbapenem-resistant *E. cloacae* and the difference in genetic affinity, which is consistent with the study of Gomez-Simmonds et al. (2016). Our study showed that ST418 is the main epidemic strain in Shenzhen in China; while in America, Central de Asturias of Spain, and the Henan province of China, the main epidemic strains were ST171, ST74, and ST120, respectively (Fernández et al., 2015; Liu et al., 2015; Gomez-Simmonds et al., 2016). We found that ST418 was genetically closer to ST127 and ST755 with START2 analysis. Studies have found that all ST418 strains produced NDM-1 carbapenemase, indicating that there might be a small outbreak of NDM-1-ST418 carbapenem-resistant *E. cloacae* in Shenzhen and Dongguan City of Guangdong province of China. In this study, ST78-NDM-1-type carbapenem-resistant *E. cloacae* was also found in Xuzhou, Xi'an, Wuhan, and Xiamen, which should be taken seriously concern.

In conclusion, our study indicates that ST418, which produces NDM-1 carbapenemase, is the main epidemic strain of carbapenem-resistant *E. cloacae* in Shenzhen and Dongguan City of China. Early detection and monitoring are necessary to prevent the further spread of the bacteria.

## Author contributions

HW conceived and designed the study. CJ and JZ wrote this paper. CJ, QW, and JZ performed the experiments. QW and JZ analyzed the data. HC, XW, and YZ assisted CJ and JZ to finish the experiments. All authors approved the final version.

### Conflict of interest statement

The authors declare that the research was conducted in the absence of any commercial or financial relationships that could be construed as a potential conflict of interest.
